# Distinct Roles of Adenosine Deaminase Isoenzymes ADA1 and ADA2: A Pan-Cancer Analysis

**DOI:** 10.3389/fimmu.2022.903461

**Published:** 2022-05-18

**Authors:** Zhao-wei Gao, Lan Yang, Chong Liu, Xi Wang, Wen-tao Guo, Hui-zhong Zhang, Ke Dong

**Affiliations:** Department of Clinical Laboratory, Tangdu Hospital, Air Force Medical University, Xi’an, China

**Keywords:** ADA1, ADA2, cancer, prognosis, immune infiltration

## Abstract

**Objective:**

Adenosine deaminase (ADA) plays an important role in immune response, which includes two isoenzymes: ADA1 and ADA2. This study aims to explore the roles of ADA1 and ADA2 in cancers.

**Methods:**

Human Protein Atlas (HPA) and Gene Expression Profiling Interactive Analysis (GEPIA2) databases were used to analyze the mRNA expression of ADA1 and ADA2 in human normal cells and tumor tissues. The enzyme assay was used to detect the ADA1 and ADA2 activities in serum from cancer patients. The Kaplan–Meier (KM) plotter was used to analyze the prognostic value of ADA1 and ADA2. TIMER2.0 was used to explore how ADA1 and ADA2 correlate with immune infiltration and immune checkpoints. cBioPortal database was used to investigate the mutations of ADA1 and ADA2. LinkedOmics was used to screen the ADA1 and ADA2 expression-related genes.

**Results:**

ADA1 was significantly increased in several tumor tissues, including cholangiocarcinoma (CHOL), lymphoid neoplasm diffuse large B-cell lymphoma (DLBC), head and neck squamous cell carcinoma (HNSC), kidney renal clear cell carcinoma (KIRC), ovarian serous cystadenocarcinoma (OV), pancreatic adenocarcinoma (PAAD), thymoma (THYM), and uterine carcinosarcoma (UCS). ADA2 expression was significantly increased in esophageal carcinoma (ESCA), glioblastoma multiforme (GBM), acute myeloid leukemia (LAML), OV, PAAD, skin cutaneous melanoma (SKCM), and stomach adenocarcinoma (STAD). There were no significant changes in serum ADA1 activities in most cancers, while serum ADA2 activities were increased in most cancers. For prognosis, high ADA1 expression was associated with the poor survival in several cancers, including esophageal squamous cell carcinoma (ESCC), HNSC, KIRC, kidney renal papillary cell carcinoma (KIRP), liver hepatocellular carcinoma (LIHC), lung adenocarcinoma (LUAD), and uterine corpus endometrial carcinoma (UCEC). However, high ADA2 expression showed a favorable prognosis in breast invasive carcinoma (BRCA), cervical squamous cell carcinoma and endocervical adenocarcinoma (CESC), HNSC, KIRC, KIRP, LUAD, OV, PAAD, sarcoma, and THYM. ADA1 showed a moderate positive correlation with multiple infiltrating immune cells in most cancers. ADA2 was positively correlated with B cells, CD8 T cells, monocytes/macrophages, and dendritic cells (DCs) and was strongly negatively correlated with myeloid-derived suppressor cells. Function analysis showed that ADA1 expression-related genes were mainly enriched in cell division biological progression. However, ADA2-related genes were mainly associated with immune response.

**Conclusion:**

As isoenzymes, ADA1 and ADA2 showed opposite prognostic values and different correlative patterns with immune infiltrating. These data demonstrated the distinct roles of ADA1 and ADA2 in cancer. ADA2 might act as a protective factor in cancer.

## Introduction

Adenosine deaminase (ADA) contains two isoenzymes: ADA1 and ADA2. ADA1 is encoded by *ADA* gene (ID: 100; location: 20q13.12). ADA2 is encoded by *ADA2* (also known as *CECR1*) gene (ID: 51816; location: 22q11.1). ADA1 and ADA2 catalyze the deamination of adenosine, which is a key immunosuppressive signal. Notably, the affinity of ADA1 for adenosine is greater than that of ADA2 (1). ADA1 is a ubiquitously expressed intracellular protein. Deficiency of ADA1 in humans results in severe combined immunodeficiency (SCID), which is characterized by profound lymphopenia, impaired differentiation, and function of T, B, and natural killer (NK) cells (2). ADA1 deficiency-induced SCID can be treated by enzyme replacement therapy (ERT) with polyethylene-glycol-modified ADA (PEG-ADA) or gene therapy (3). Moreover, elevated ADA1 activity in erythrocytes has been considered the biomarker of Diamond-Blackfan anemia (4).

In contrast with ADA1, ADA2 is a plasma protein secreted primarily by monocytes and macrophages (5). ADA2 deficiency can lead to a range of clinical symptoms in patients, including childhood-onset stroke, systemic vasculitis, variable immunodeficiency, and hematologic defects (6–9). Moreover, studies showed that ADA2 activities were increased in tubercular pleural effusion, tubercular cerebrospinal fluid, and several autoimmune diseases, such as systemic lupus erythematosus (SLE), autoimmune liver disease (AILD), and macrophage activation syndrome of systemic juvenile idiopathic arthritis (sJIA) (10–13).

A multitude of immunosuppressive mechanisms occurring in the tumor microenvironment (TME) has been identified, including the accumulation of extracellular adenosine (14). Thus, as the enzymes that catalyze adenosine degradation, ADA1 and ADA2 may play important roles during tumor development. Several studies have shown the change of ADA activities in serum and tumor tissues of breast cancer patients (15, 16). A recent study showed that PEGylated ADA2 injection inhibited the growth of several solid tumors (including colon and breast tumors) and lung metastasis in tumor-bearing mice models, which indicated the potential value of ADA in cancer therapy (17). However, the studies about ADA in cancers are still few, and the roles of ADA1 and ADA2 in pan-cancer are still unclear. As isoenzymes, there is a variety of differences between ADA1 and ADA2. Here, we conducted a pan-cancer analysis to illustrate the potential function of ADA1 and ADA2 in cancers. Moreover, by using clinical samples, we investigated the change of serum ADA1 and ADA2 activities in various cancers.

## Material and Methods

### Expression Profile Analysis of ADA1 and ADA2

Human Protein Atlas (HPA; https://www.proteinatlas.org/) database was used to illustrate ADA1 and ADA2 mRNA distribution among different cells. Gene Expression Profiling Interactive Analysis (GEPIA2, http://gepia2.cancer-pku.cn/#index) was used to analyze the difference in ADA1 and ADA2 mRNA expression between tumor and non-tumor control tissues based on The Cancer Genome Atlas (TCGA) and Genotype-Tissue Expression (GTEx) databases. |Log2FC| > 1, p < 0.05 was seen as statistical significance.

### Prognostic Value Analysis of ADA1 and ADA2

The Kaplan–Meier (KM) plotter was used to analyze the prognostic value of ADA1 and ADA2 in pan-cancer. The “auto select best cutoff” model was chosen, which means that all possible cutoff values were computed, and the best-performing threshold was used as the cutoff. R forestplot and survival curves were used to display the results.

### Immune Infiltration Analysis

Tumor Immune Estimation Resource 2.0 (TIMER2.0; http://timer.cistrome.org/) was used to investigate the molecular characterization of tumor-immune interactions. “Immune-Gene” module was used to evaluate the relationship between ADA1 and ADA2 levels and the immune cells (including B cells, T cells, monocytes/macrophages, neutrophils, dendritic cells, NK cells, etc.) and infiltrating levels in pan-cancer. The “Exploration-Gene_Corr” module was used to analyze the correlation between ADA1, ADA2, and immune checkpoints.

### Mutation Profiles of ADA1 and ADA2

The cBioPortal for cancer genomics (http://www.cbioportal.org) is an open-access repository of cancer genomics datasets. The cBioPortal was used to investigate the alteration landscapes of ADA1 and ADA2 in pan-cancer.

### Gene Enrichment Analysis

ADA1 and ADA2 expression-related genes (correlation coefficient |r| > 0.4, p < 0.001) were screened by using LinkedOmics based on TCGA data. Then, function analysis was performed by using DAVID Bioinformatics Resources (https://david.ncifcrf.gov/tools.jsp) (18). R Bubble diagrams were used for visualization of the enriched Gene Ontology (GO) term and Kyoto Encyclopedia of Genes and Genomes (KEGG) pathway.

### Clinical Sample Collection

The detailed information of the clinical sample is listed in **Supplementary Table 2**. All diagnoses were confirmed by pathology. Healthy subjects were recruited as the control group. Peripheral blood from cancer patients was collected and subjected to a 4,000-rpm centrifuge for 5 min at 4°C to obtain serum. Serum was stored at −20°C. Ethical approval was obtained from the ethics committee of Tangdu Hospital, The Fourth Military Medical University. Waiver of informed consent would not affect the rights and interests of the subjects. Thus, informed consent was not required in this study.

### Serum Adenosine Deaminase Activity Detection

In the previous description (13), serum total ADA activities were measured by using the enzymatic kit (Shanghai Kehua Bio-engineering, Shanghai, China), adapted to the automated biochemistry analyzer (Hitachi 7600, Tokyo, Japan). The serum ADA2 activities were measured in the presence of 0.1 mM of erythro-9-(2-hydroxy-3-nonyl) adenine (EHNA), which is a selective inhibitor of ADA1. The serum ADA1 activity was calculated by subtracting the ADA2 activity from the total ADA activity. The results were expressed as units per liter (U/L).

### Statistical Analysis

Serum ADA1 and ADA2 levels were expressed as the mean (SD). Student’s t-test test was used to analyze the difference in serum ADA activities between patients and healthy controls. Pearson’s correlation analyses were used to gauge the degree of correlation between genes expression. p < 0.05 was considered to be statistically significant.

## Results

### ADA1 and ADA2 Expression Profiles in Tumors

**Figure 1:**
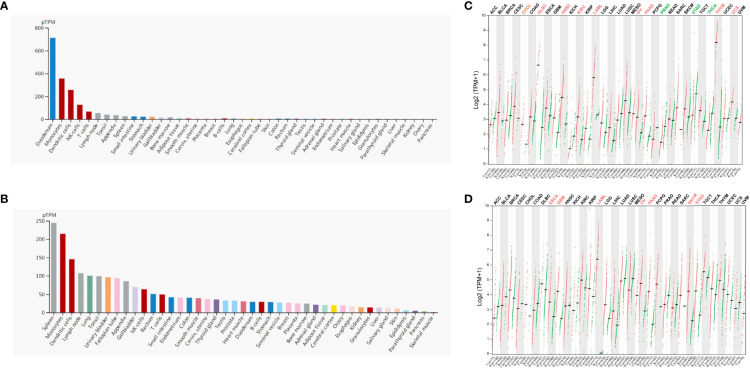
ADA1 and ADA2 expression profiles in human tissues. **(A, B)** HPA database showed the ADA1 and ADA2 expression in normal human tissues. **(C, D)** Comparisons of ADA1 and ADA2 expression levels between tumor and non-tumor control tissues based on TCGA and GTEx database (black, expression levels showed no significant difference between tumor and normal; red, expression levels were increased in tumor; green, expression levels were decreased in tumor). HPA, Human Protein Atlas; TCGA, The Cancer Genome Atlas; GTEx, Genotype-Tissue Expression.

Firstly, we evaluated the expression profile of ADA and ADA2 in human tissues and cells based on the HPA database. The results showed that ADA1 expression was enriched in the duodenum, monocytes, dendritic cells (DCs), NK cells, and T cells, and ADA2 was enriched in the spleen, monocytes, DCs, lymph nodes, and lung (**Figures 1A, B**). Furthermore, we investigated the differential expression of ADA1 and ADA2 between tumor and normal tissues. ADA1 was significantly increased in cholangiocarcinoma (CHOL), lymphoid neoplasm diffuse large B-cell lymphoma (DLBC), head and neck squamous cell carcinoma (HNSC), kidney renal clear cell carcinoma (KIRC), acute myeloid leukemia (LAML), ovarian serous cystadenocarcinoma (OV), pancreatic adenocarcinoma (PAAD), thymoma (THYM), and uterine carcinosarcoma (UCS) while decreased in prostate adenocarcinoma (PRAD), stomach adenocarcinoma (STAD), and thyroid carcinoma (THCA) (**Figure 1C**). ADA2 was significantly increased in esophageal carcinoma (ESCA), glioblastoma multiforme (GBM), LAML, OV, PAAD, skin cutaneous melanoma (SKCM), and STAD (**Figure 1D**). Notably, in LAML, OV, and PAAD tumor tissues, both ADA1 and ADA2 were increased, while in STAD tumor tissues, the expression changes of ADA1 and ADA2 were opposite.

### The Potential Prognostic Value of ADA1 in Cancers

**Figure 2:**
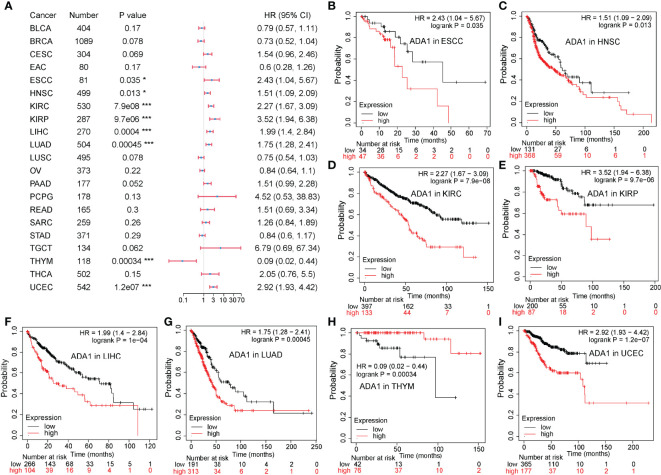
Prognostic value of ADA1 in different cancers. **(A)** Forest plots showed the relation between ADA1 expression and OS of cancer patients. **(B–I)** Survival curves of ESCC, HNSC, KIRC, KIRP, LIHC, LUAD, THYM, and UCEC. *p < 0.05, ***p < 0.001. OS, overall survival; ESCC, esophageal squamous cell carcinoma; HNSC, head and neck squamous cell carcinoma; KIRC, kidney renal clear cell carcinoma; KIRP, kidney renal papillary cell carcinoma; LIHC, liver hepatocellular carcinoma; LUAD, lung adenocarcinoma; THYM, thymoma; UCEC, uterine corpus endometrial carcinoma.

To evaluate the potential prognostic value of ADA1 and ADA2, KM plotter was used to analyze the correlation between patients’ overall survival (OS) and expression of ADA1 and ADA2. As shown in **Figure 2**, high ADA1 expression was associated with the poor OS in several cancers, including esophageal squamous cell carcinoma [ESCC; HR (95% CI) = 2.43 (1.04–5.67), p = 0.035], HNSC [HR (95% CI) = 1.51 (1.09–2.09), p = 0.013], KIRC [HR (95% CI) = 2.27 (1.67–3.09), p = 7.9e−08], kidney renal papillary cell carcinoma [KIRP; HR (95% CI) = 3.52 (1.94–6.38), p = 9.7e−06], liver hepatocellular carcinoma [LIHC; HR (95% CI) = 1.99 (1.4–2.84), p = 4e−04], lung adenocarcinoma [LUAD; HR (95% CI) = 1.75 (1.28–2.41), p = 4.5e−04], and uterine corpus endometrial carcinoma [UCEC; HR (95% CI) = 2.92 (1.93–4.42), p = 1.2e−07]. However, high ADA1 expression significantly related to a better prognosis in THYM [HR (95% CI) = 0.09 (0.02–0.44), p = 3.4e−04]. These results indicated that ADA1 plays different roles in THYM and other cancers.

### The Potential Prognostic Value of ADA2 in Cancers

**Figure 3:**
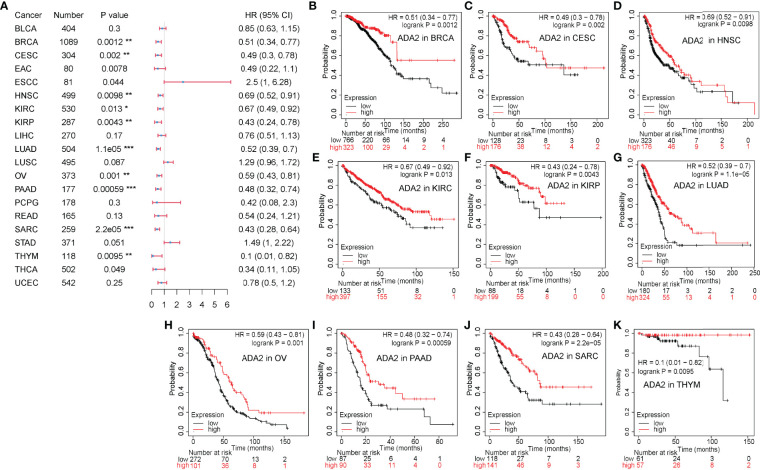
Prognostic value of ADA2 in different cancers. **(A)** Forest plots showed the relation between ADA2 expression and OS of cancer patients. **(B–K)** Survival curves of BRCA, CESC, HNSC, KIRC, KIRP, LUAD, OV, PAAD, SARC, and THYM. *p < 0.05, **p < 0.01, ***p < 0.001. OS, overall survival; BRCA, breast invasive carcinoma; CESC, cervical squamous cell carcinoma and endocervical adenocarcinoma; HNSC, head and neck squamous cell carcinoma; KIRC, kidney renal clear cell carcinoma; KIRP, kidney renal papillary cell carcinoma; LUAD, lung adenocarcinoma; OV, ovarian serous cystadenocarcinoma; PAAD, pancreatic adenocarcinoma; SARC, sarcoma; THYM, thymoma.

On the contrary, as shown in **Figure 3**, in a variety of cancers, patients with high ADA2 expression showed a favorable prognosis, including breast invasive carcinoma [BRCA; HR (95% CI) = 0.51 (0.34–0.77), p = 0.0012], cervical squamous cell carcinoma and endocervical adenocarcinoma [CESC; HR (95% CI) = 0.49 (0.3–0.78), p = 0.002], HNSC [HR (95% CI) = 0.69 (0.52–0.91), p = 0.0098], KIRC [HR (95% CI) = 0.67 (0.49–0.92), p = 0.013], KIRP [HR (95% CI) = 0.43 (0.24–0.78), p = 0.0043], LUAD [HR (95% CI) = 0.52 (0.39–0.7), p = 1.1e−05], OV [HR (95% CI) = 0.59 (0.43–0.81), p = 0.001], PAAD [HR (95% CI) = 0.38 (0.32–0.74), p = 5.9e−04], sarcoma [SARC; HR (95% CI) = 0.43 (0.28–0.64), p = 2.2e−05], and THYM [HR (95% CI) = 0.1 (0.01–0.82), p = 0.0095]. Taken together, these results demonstrated the different prognostic value between ADA1 and ADA2 in several types of cancers.

### The Correlation Between Immune Infiltration and ADA1 and ADA2

**Figure 4:**
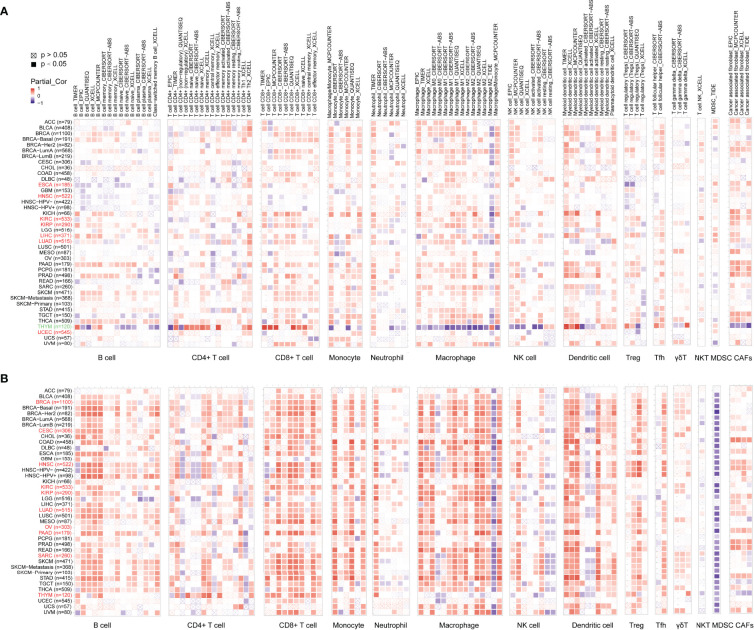
Associations of ADA1 and ADA2 expression with immune infiltration. **(A)** The correlations between ADA1 expression and immune infiltration in cancers. **(B)** The correlations between ADA2 expression and immune infiltration in cancers.

To explore whether ADA was involved in the process of tumor-immune infiltration, we employed TIMER2.0 to analyze the correlation between tumor-infiltrating immune cells and the expression of ADA1 and ADA2. In most tumors, we observed a moderate positive correlation between ADA1 and multiple infiltrating immune cells (**Figure 4A**), including B cells, T cells, monocytes/macrophages, DCs, NK cells, and cancer-associated fibroblasts (CAFs). However, in THYM, ADA1 was negatively correlated with monocytes/macrophages, NK cells, and CAFs and was positively correlated with CD4^+^ T, CD8^+^ T cells, and DCs. These results indicated the potential mechanism of the different prognostic values of ADA1 in THYM and other tumors.

As shown in **Figure 4B**, the relationship between ADA2 and infiltrating immune cells was consistent across pan-cancer. Moreover, compared to ADA1, the correlation coefficient between ADA2 and infiltrating cells was higher. ADA2 was strongly positively correlated with B cells, CD8 T cells, monocytes/macrophages, and DCs. However, a strong negative correlation between ADA2 and myeloid-derived suppressor cells (MDSCs) was observed. These results suggested that decreased MDSC infiltration might be associated with the favorable prognosis of patients with higher ADA2 expression.

### The Relationship Between Immune Checkpoints and ADA1 and ADA2

**Figure 5:**
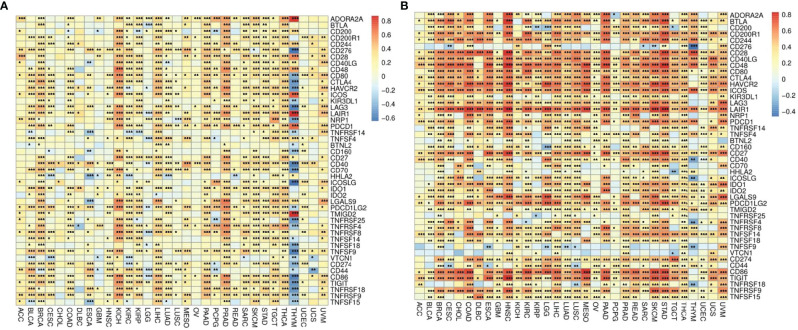
The correlations between ADA1, ADA2, and immune checkpoints. **(A)** The correlations between ADA1 and confirmed immune checkpoints in multiple cancers. **(B)** The correlations between ADA2 and confirmed immune checkpoints in multiple cancers. *p < 0.05, **p < 0.01, ***p < 0.001.

The correlations between ADA1 and immune checkpoints are shown in **Figure 5A**. First of all, in THYM, *ADA1* is positively related to *TMIGD2*, *LAIR1*, *ADORA2A*, *ICOS*, and *PDCD1* while negatively related to *ICOSLG*, *TNFRSF25*, *TNFSF18*, *CD40*, *LAG3*, *TNFSF9*, *CD80*, *CD276*, *TNFRSF18*, etc. Secondly, in KIRC and PRAD, there was a strong positive correlation between ADA1 and most immune checkpoints. Moreover, in most cancers except GBM, LGG, CHOL, UCEC, HNSC, lung squamous cell carcinoma (LUSC), and OV, there was a moderate positive correlation between *ADA1* and immune checkpoints. Notably, compared to that in other cancers, the correlation profile between *ADA1* and immune checkpoints was significantly different in THYM.

*ADA2* (**Figure 5B**), in most cancers, positively correlated with *LGALS9*, *CD80*, *CTLA4*, *LAG3*, *TNFRSF9*, *PDCD1LG2*, *CD274*, *IDO1*, *TNFSF14*, *CD244*, *CD200R1*, *CD28*, *BTLA*, *PDCD1*, *CD40LG*, *ICOS*, *LAIR1*, *HAVCR2*, *CD86*, *CD48*, *CD27*, and *TIGIT*. Moreover, *ADA2* negatively correlated with *CD276*, *TNFSF9*, and *VTCN1* in most cancers. Compared with *ADA1*, *ADA2* showed a higher correlation with immune checkpoints.

### The Mutation Profile of ADA1 and ADA2 in Cancers

**Figure 6:**
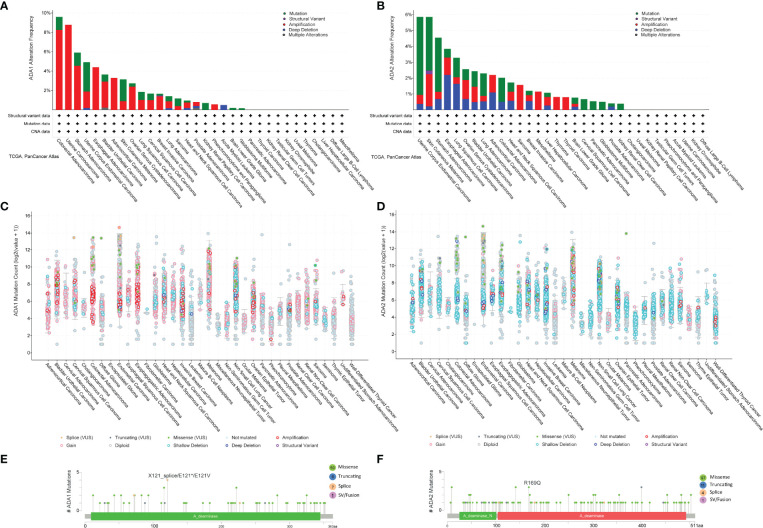
ADA1 and ADA2 mutation landscape. **(A, B)** ADA1 and ADA2 mutation frequency in multiple TCGA pan-cancer studies according to the cBioPortal database. **(C, D)** The general mutation count of ADA1 and ADA2 in various TCGA cancer types by the cBioPortal database. **(E, F)** Mutation diagram of ADA1 and ADA2 in different cancer types across protein domains. TCGA, The Cancer Genome Atlas.

We employed the cBioPortal to investigate the mutation profile of *ADA1* and *ADA2*. In summary, the alteration frequency of *ADA1* and *ADA2* was lower than 5% in most cancers. As shown in **Figure 6A**, the top 5 cancer types that contained *ADA1* alteration were colorectal adenocarcinoma (COADREAD), UCS, STAD, UCEC, and ESCA. The major alteration type of *ADA1* gene was amplification. Furthermore, for *ADA2* alteration, the top 5 cancers were UCEC, SKCM, STAD, ESCA, and LUSC (**Figure 6B**). The major alteration type of *ADA2* gene was a mutation. Deep deletion of *ADA1* was rare in most cancers, while deep deletion of *ADA2* was found in most cancers (**Figures 6A–D**). As shown in **Figure 6E**, a total of 73 mutations were found in *ADA1* (including 59 missense, 6 truncating, 7 splice, and 1 fusion). For *ADA2* (**Figure 6F**), a total of 113 mutations were found (including 97 missense, 11 truncating, 4 splice, and 1 fusion).

### Enrichment Analysis of ADA1 and ADA2 Expression-Related Genes in Lung Adenocarcinoma, Kidney Renal Clear Cell Carcinoma, and Kidney Renal Papillary Cell Carcinoma

**Figure 7:**
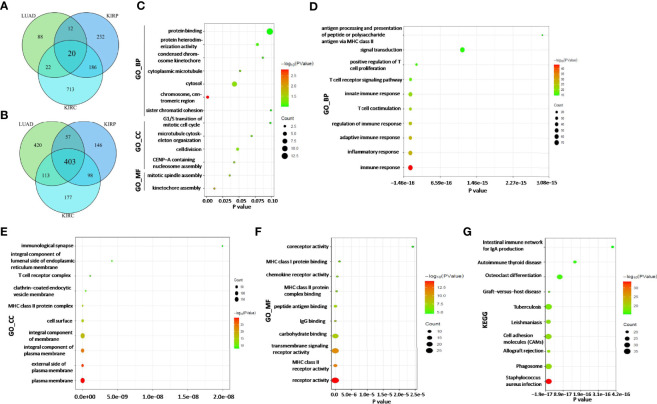
ADA1 and ADA2 expression-related genes and function analysis. **(A)** The shared gene signature positively correlated to ADA1 expression among LUAD, KIRP, and KIRC. **(B)** The shared gene signature positively correlated to ADA2 expression among LUAD, KIRP, and KIRC. **(C)** Function enrichment analysis of shared ADA1-related genes (n = 20). **(D–G)** Function enrichment analysis of shared ADA2-related genes (n = 403). TCGA, The Cancer Genome Atlas; LUAD, lung adenocarcinoma; KIRP, kidney renal papillary cell carcinoma; KIRC, kidney renal clear cell carcinoma.

According to the survival analysis, we observed contradictory prognostic values of ADA1 and ADA2 in LUAD, KIRC, and KIRP. Thus, we further screened the ADA1 and ADA2 expression-associated genes in these cancers and performed a functional analysis of these genes. By using LinkedOmics, the positively correlated genes with ADA1 and ADA2 in LUAD, KIRC, and KIRP were screened (correlation coefficient r > 0.4, p < 0.001). Among ADA1 positively related genes in three cancers, there were 20 shared genes (**Figure 7A**). For ADA2 positively related genes, there were 403 shared genes (**Figure 7B**). These shared genes are listed in **Supplementary Table 2**. *NKG7* is the only gene that positively related to both ADA1 and ADA2 in three cancers.

Next, function enrichment analysis was performed to investigate the biological characteristics associated with ADA1-related genes (**Supplementary Table 2**). As shown in **Figure 7C**, for biological process (GO_BP), the ADA1 expression correlated genes were mainly related to cell division, including kinetochore assembly, mitotic spindle assembly, and CENP-A containing nucleosome assembly. However, as shown in **Figures 7D–G**, ADA2 correlated genes were mainly associated with immune response, including inflammatory response, innate and adaptive immune response, T-cell costimulation, positive regulation of T-cell proliferation, etc. GO_MF (molecular function) analysis for ADA2 correlated genes showed enrichment in MHC class II receptor activity, IgG binding, peptide antigen binding, MHC class II protein complex binding, etc. Moreover, KEGG pathway analysis showed that ADA2 correlated genes were related to *Staphylococcus aureus* infection, phagosome, cell adhesion molecules, osteoclast differentiation, etc. Taken together, the different functions of ADA1 correlated genes and ADA2 correlated genes might contribute to the different prognostic values of ADA1 and ADA2.

### The Serum ADA1 and ADA2 Activities in Cancers

**Figure 8:**
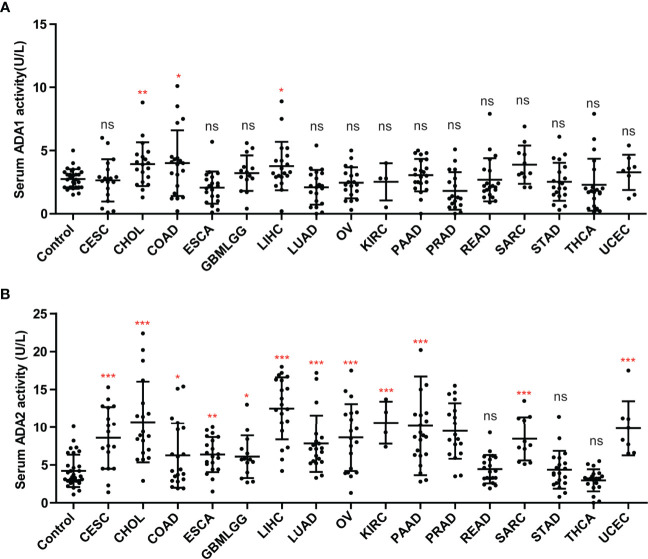
The activities of ADA1 and ADA2 in serum from cancer patients. **(A)** Serum ADA1 activity in cancers. **(B)** Serum ADA2 activity in cancers. *p < 0.05, **p < 0.01, ***p < 0.001. ns, no significant.

We investigated the ADA1 and ADA2 activities in serum from cancer patients, including CESC, CHOL, colon adenocarcinoma (COAD), ESCA, GBM, LIHC, LUAD, OV, KIRC, KIRP, PAAD, PRAD, rectal adenocarcinoma (READ), SARC, STAD, THCA, and UCEC. Compared to controls, there were no significant changes in serum ADA1 activities in most cancers, except in CHIL, COAD, and LIHC (**Figure 8A**). However, serum ADA2 activities were significantly increased in most cancers, except in READ, STAD, and THCA (**Figure 8B**).

## Discussion

Adenosine concentration *in vivo* is regulated by enzyme cascade, as follows: ATP/ADP is degraded into AMP, which is catalyzed by CD39 (also known as E-NTPDase1); AMP is degraded into adenosine, which is catalyzed by CD73 (ecto-5′-nucleotidase, *NT5E*); adenosine is degraded into inosine, which is catalyzed by ADA. Adenosine acts as an immunosuppressive signal in humans (19). Thus, targeting the adenosine pathway has been considered a promising therapeutic strategy for cancer. Several clinical studies targeting CD39, CD73, and adenosine receptors are ongoing. Some beneficial outcomes for both mono-target therapy and combinations have been reported (20, 21). However, the function and clinical value of ADA in cancers have not been well analyzed, including ADA1 and ADA2.

As the isoenzymes, ADA1 and ADA2 have ADA activities. However, increasing evidence has shown that there were several different features and functions between ADA1 and ADA2. Firstly, in the different locations of ADA1 and ADA2, ADA1 is mostly expressed intracellularly, while ADA2 is mostly secreted extracellularly. Thus in plasma or serum, total ADA activity is mainly constituted by ADA2 activity and is highly positively correlated with ADA2 activity (22, 23). Secondly, ADA1 shows a higher affinity toward adenosine and higher catalytic activity than ADA2. Thirdly, Human ADA1 and ADA2 could bind to different subsets of immune cells (24). ADA1 could bind to the surface of immune cell subtypes *via* dipeptidyl peptidase IV (CD26). ADA2 belonged to the ADA deaminase growth factor family and could bind to immune cell subtypes that do not express CD26, *via* proteoglycans or other unknown receptors. A study by Zavialov et al. showed that ADA2 induces the differentiation of monocytes into macrophages and stimulates the proliferation of T helper cells and macrophages (25). Fourthly, the diseases induced by the deficiency of ADA1 and ADA2 were different; ADA-SCID is a well-known ADA1 deficiency that causes diseases that could be cured by ADA1 replacement therapy (26). However, ADA2 deficiency was associated with a spectrum of vascular and inflammatory phenotypes, including early-onset recurrent stroke and systemic vasculopathy (27, 28). Taken together, because of these differences between ADA1 and ADA2, we presume that ADA1 and ADA2 may play different roles in cancers.

In this study, we investigated the distinct roles of ADA1 and ADA2 in pan-cancer. In general, the mutation frequency of ADA1 and ADA2 in cancers was low. It may be understandable because the ADA1/ADA2 mutations usually cause severe disease in children. The highlighted results of this study are as follows: firstly, the ADA1 and ADA2 showed opposite prognostic values in several types of cancers. In cancers with a significant association with ADA1 or ADA2 levels, higher ADA1 showed a poor prognosis (except for THYM), while higher ADA2 was associated with better survival. Recently, a study by Wang et al. found that PEGylated ADA2 injection could inhibit tumor growth in colon and breast cancer murine models (17). These data indicated that ADA2 might be a protected factor in several tumor types (BRCA, CESC, HNSC, KIRC, KIRP, LUAD, OV, PAAD, and THYM). A study by Lee et al. showed that ADA2 levels could be stimulated by cytokines, such as IFNγ, IL-12, IL-18, and TNF-α (13). IFNγ, IL-12, IL-18, and TNF-α have shown antitumor effects (29–31). Thus, the correlation between ADA2 and these cytokines might be a potential mechanism for ADA2’s favorable prognostic value. A study by Wang et al. has shown the antitumor effects of ADA2 protein injection for several tumors (17). Based on the analysis of this study, the potential antitumor effects of ADA2 proteins or ADA2 agonists in more cancers needed to be investigated. Moreover, to better understand the different roles of ADA1 and ADA2, the effect of ADA1 protein and ADA1 inhibitor EHNA on tumors growth and metastasis should be further studied. Nakajima’s study has shown that EHNA exhibits a potent anticancer effect against malignant pleural mesothelioma (32).

Secondly, in the different correlation profiles between infiltrating immune cells and ADA1 or ADA2, ADA1 showed a moderate positive correlation with multiple infiltrating immune cells in most cancers except THYM. ADA2 was strongly positively correlated with B cells, CD8 T cells, monocytes/macrophages, and DCs and was strongly negatively correlated with MDSCs. The correlations between ADA1/ADA2 and immune checkpoints also supported this conclusion. Accumulated evidence has shown that MDSCs contributed to cancer progression *via* various mechanisms, including hampering antitumor-immune responses (33, 34). Thus, the favorable prognostic value of ADA2 could be explained, at least in part, by the negative correlation between ADA2 and MDSC infiltration.

Thirdly, we screened the genes positively correlated with ADA1 or ADA2 in LUAD, KIRP, and KIRC. Among 20 ADA1 correlated genes and 403 ADA2 correlated genes, there was only one shared gene, *NKG7*. Furthermore, function analysis showed that these 20 ADA1-related genes were mainly enriched in cell division biological progression, including kinetochore assembly and mitotic spindle assembly. However, these 403 ADA2-related genes were mainly associated with immune response, including inflammatory response, innate and adaptive immune response, T-cell costimulation, and positive regulation of T-cell proliferation. These data indicated the potential mechanism of distinct roles for ADA1 and ADA2 in cancers.

Interestingly, high ADA1 expression was associated with better survival in THYM patients, which was in contrast to ADA1’s prognostic value in other cancers. Moreover, between THYM and other cancers, the correlation profile between ADA1 and infiltrating immune cells or immune checkpoints also showed significant differences. Studies have shown that ADA1 was critical for thymus development, and ADA1 deficiency promoted thymic T-cell apoptosis (35–37). ADA1 deficiency caused SCID characterized by a deficiency of T cells caused by arrested development in the thymus. The unique need for ADA1 in the thymus might be a reason for the different roles of ADA1 between THYM and other cancers.

Notably, the ADA1 and ADA2 levels in tumor tissues and serum from cancer patients do not have a corresponding relationship. This difference might be due to the different locations of ADA1 (intracellular) and ADA2 (extracellular). As a secretory protein, ADA2 was increased in serum in most cancers. However, the prognostic value of serum ADA2 has not been revealed. Serum ADA2 is easy to test; thus, there is a potential application value for the study on the clinical significance of serum ADA2.

## Conclusion

In conclusion, we applied integrated bioinformatics analysis to demonstrate the roles of ADA1 and ADA2 in cancers. We concluded that ADA1 was a poor prognostic marker in several cancers, while ADA2 was a favorable prognostic marker. The opposite prognostic value for ADA1 and ADA2 may be explained by their different correlation with immune infiltration and immune checkpoints. Thus, although the ADA isoenzymes ADA1 and ADA2 play different roles in cancer development, the functions and mechanisms for ADA1 and ADA2 need to be validated by further studies.

## Data Availability Statement

The original contributions presented in the study are included in the article/**Supplementary Material**. Further inquiries can be directed to the corresponding author.

## Ethics Statement

Ethical approval was obtained from the ethics committee of Tangdu Hospital, The Fourth Military Medical University. Written informed consent for participation was not required for this study in accordance with the national legislation and the institutional requirements.

## Author Contributions

Z-wG and KD conceived and designed this study. Z-wG and LY performed the bioinformatics analyses and visualization. CL, W-tG, and XW collected the data and performed the statistical analysis. Z-wG and CL wrote the original draft. H-zZ and KD revised the manuscripts. All authors revised and approved the final manuscript.

## Funding

This study was supported by the National Natural Science Foundation of China (81702732) and Scientist Fund of Tangdu Hospital (2021SHRC004).

## Conflict of Interest

The authors declare that the research was conducted in the absence of any commercial or financial relationships that could be construed as a potential conflict of interest.

## Publisher’s Note

All claims expressed in this article are solely those of the authors and do not necessarily represent those of their affiliated organizations, or those of the publisher, the editors and the reviewers. Any product that may be evaluated in this article, or claim that may be made by its manufacturer, is not guaranteed or endorsed by the publisher.

## Supplementary Material

Supplementary Table 1 The detail information of serum sample.

Supplementary Table 2 The list of ADA1 and ADA2 expression related genes and function enrichment of these genes.

## Abbreviations

ACC, adrenocortical carcinoma; BLCA, bladder urothelial carcinoma; BRCA, breast invasive carcinoma; CESC, cervical squamous cell carcinoma and endocervical adenocarcinoma; CHOL, cholangiocarcinoma; COAD, colon adenocarcinoma; DLBC, lymphoid neoplasm diffuse large B-cell lymphoma; ESCA, esophageal carcinoma; GBM, glioblastoma multiforme; HNSC, head and neck squamous cell carcinoma; KICH, kidney chromophobe; KIRC, kidney renal clear cell carcinoma; KIRP, kidney renal papillary cell carcinoma; LAML, acute myeloid leukemia; LGG, brain lower-grade glioma; LIHC, liver hepatocellular carcinoma; LUAD, lung adenocarcinoma; LUSC, lung squamous cell carcinoma; MESO, mesothelioma; OV, ovarian serous cystadenocarcinoma; PAAD, pancreatic adenocarcinoma; PCPG, pheochromocytoma and paraganglioma; PRAD, prostate adenocarcinoma; READ, rectal adenocarcinoma; SARC, sarcoma; SKCM, skin cutaneous melanoma; STAD, stomach adenocarcinoma; TGCT, testicular germ cell tumors; THCA, thyroid carcinoma; THYM, thymoma; UCEC, uterine corpus endometrial carcinoma; UCS, uterine carcinosarcoma; UVM, uveal melanoma; HPA, Human Protein Atlas; GEPIA2, Gene Expression Profiling Interactive Analysis.
